# Persistent elevation of lysophosphatidylcholine promotes radiation brain necrosis with microglial recruitment by P2RX4 activation

**DOI:** 10.1038/s41598-022-12293-3

**Published:** 2022-05-24

**Authors:** Natsuko Kondo, Yoshinori Sakurai, Takushi Takata, Kuniyuki Kano, Kyo Kume, Munetoshi Maeda, Nobuhiko Takai, Shugo Suzuki, Fumihiro Eto, Kenji Kikushima, Hideki Wanibuchi, Shin-Ichi Miyatake, Takayuki Kajihara, Shoji Oda, Mitsutoshi Setou, Junken Aoki, Minoru Suzuki

**Affiliations:** 1 Particle Radiation Oncology Center, Institute for Integrated Radiation and Nuclear Science, Kyoto University, Osaka, 590-0494 Japan; 2 Department of Health Chemistry, Graduate School of Pharmaceutical Sciences, The University of Tokyo, Tokyo, 113-0033 Japan; 3 Proton Medical Research Division, Research and Development Department, The Wakasa Wan Energy Research Center, Fukui, 914-0135 Japan; 4 Department of Analytical Chemistry, Faculty of Pharmaceutical Sciences, Nagasaki International University, Nagasaki, 859-3298 Japan; 5 Department of Molecular Pathology, Osaka Metropolitan University Graduate School of Medicine, Osaka, 545-8585 Japan; 6 Department of Cellular and Molecular Anatomy and International Mass Imaging Center, Hamamatsu University School of Medicine, Shizuoka, 431-3192 Japan; 7 Cancer Center, Osaka Medical and Pharmaceutical University, Osaka, 569-8686 Japan; 8 Department of Integrated Biosciences, Graduate School of Frontier Sciences, The University of Tokyo, Chiba, 277-8562 Japan

**Keywords:** Neuroscience, Neuroimmunology

## Abstract

Brain radiation necrosis (RN) or neurocognitive disorder is a severe adverse effect that may occur after radiation therapy for malignant brain tumors or head and neck cancers. RN accompanies inflammation which causes edema or micro-bleeding, and no fundamental treatment has been developed. In inflammation, lysophospholipids (LPLs) are produced by phospholipase A_2_ and function as bioactive lipids involved in sterile inflammation in atherosclerosis or brain disorders. To elucidate its underlying mechanisms, we investigated the possible associations between lysophospholipids (LPLs) and RN development in terms of microglial activation with the purinergic receptor P2X purinoceptor 4 (P2RX4). We previously developed a mouse model of RN and in this study, measured phospholipids and LPLs in the brains of RN model by liquid chromatography tandem mass spectrometry (LC–MS/MS) analyses. We immune-stained microglia and the P2RX4 in the brains of RN model with time-course. We treated RN model mice with ivermectin, an allosteric modulator of P2RX4 and investigate the effect on microglial activation with P2RX4 and LPLs’ production, and resulting effects on overall survival and working memory. We revealed that LPLs (lysophosphatidylcholine (LPC), lysophosphatidyl acid, lysophosphatidylserine, lysophosphatidylethanolamine, lysophosphatidylinositol, and lysophosphatidylglycerol) remained at high levels during the progression of RN with microglial accumulation, though phospholipids elevations were limited. Both microglial accumulation and activation of the P2RX4 were attenuated by ivermectin. Moreover, the elevation of all LPLs except LPC was also attenuated by ivermectin. However, there was limited prolongation of survival time and improvement of working memory disorders. Our findings suggest that uncontrollable increased LPC, even with ivermectin treatment, promoted the development of RN and working memory disorders. Therefore, LPC suppression will be essential for controlling RN and neurocognitive disorder after radiation therapy.

## Introduction

Brain radiation necrosis (RN) and neurocognitive disorders are late adverse events that are often accompanied by chronic inflammation following radiotherapy for malignant brain tumors or head and neck cancers^1^. Neurocognitive disorders lower the quality of life, especially in child patients when they occur in memory. Many studies have confirmed that bevacizumab effectively relieves RN-induced brain edema symptoms and improves patients’ Karnofsky performance status scores and brain necrosis imaging. However, necrosis is irreversible, and hypoxia and ischemia localized in the area of brain necrosis can lead to brain RN recurrence after bevacizumab is discontinued^2^; as yet, no fundamental treatment for RN has been established.

In inflammation, phospholipase A_2_ (PLA_2_) catalyzes the hydrolysis of membrane glycerophospholipids at the sn-2 ester bond position, leading to the production of free fatty acids and lysophospholipids (LPLs). LPLs function as bioactive lipids, and recent studies have implicated LPLs in specific receptor-mediated signaling^3^. In addition, irradiation with either gamma rays or particle radiation increases phospholipids (phosphatidylethanolamine [PE], phosphatidylserine [PS], or phosphatidylcholine [PC]) and some lysophosphatidylcholines (LPCs), which is suggestive of a phospholipid metabolic abnormality^4–6^. In the present study, we used a brain RN mouse model that we established using proton beam irradiation^7,8^. This mouse model develops RN 6–8 months after localized irradiation to one hemisphere of the brain at a dose of 60 Gy, and the pathology recapitulates human brain RN accompanied by chronic inflammation. On the other hand, we confirmed neither X-ray nor proton irradiation to the right hemisphere of mouse brain at a dose of 40 Gy induced RN. Therefore, we also used the X-ray irradiation group as a representative which do not develop RN.

Microglia are involved in neuroinflammatory diseases, such as neuropathic pain^9^, Alzheimer’s disease^10^, and cerebral infarction^11^, through activation by LPLs such as lysophosphatidyl acid (LPA)^8^ or LPC^11^. LPC possesses proinflammatory properties through binding to cell surface receptors, such as G protein-coupled receptor 132 (G2A)^12^ and P2X purinoceptor 7 (P2RX7)^13^, on microglia.

Ivermectin, an allosteric modulator of P2X purinoceptor 4 (P2RX4) signaling, affects microglial polarization to favor the switch from a proinflammatory to an anti-inflammatory phenotype, potentiates myelin phagocytosis, promotes the remyelination response, and ameliorates clinical signs of experimental autoimmune encephalomyelitis (EAE) in animal models^14^. Ivermectin has also been reported to extend the lifespan of a transgenic mouse model of amyotrophic lateral sclerosis (ALS)^15^.

Therefore, we first investigated whether LPLs are involved in the pathogenesis of RN and in microglial activation in RN. We revealed a persistent elevation of LPLs and microglial activation in our RN model. Next, we analyzed the effects of continuous ivermectin treatment on LPL production and microglial activation through P2RX4 in this RN model. Ivermectin repressed the production of all LPLs except LPC, and reduced microglial activation through P2RX4. However, ivermectin induced only a slight, if any, and not significant prolongation of survival time and changes in working memory disorders in the RN mouse model; these findings were probably related to the persistent elevation of LPC.

## Methods

### Animals

Mice were kept under conventional housing conditions (24 °C, 55% ± 10% humidity, and a 12-h day/night cycle) at animal facilities in the Institute for Integrated Radiation and Nuclear Science, Kyoto University. All methods were performed in accordance with the institutional guidelines and regulations of the animal care of Kyoto University and the Wakasa Wan Energy Research Center. The study was performed in accordance with ARRIVE guidelines (https://arriveguidelines.org).

### Irradiation to the right hemisphere of mouse brain

The RN model was induced in 10- to 12-week-old female C57Bl/6 mice using proton beam irradiation that was produced by a synchrotron accelerator at the Wakasa Wan Energy Research Center. Irradiation was applied to an area of the right hemisphere (10 mm in height, 5 mm in width, and 4 mm in depth) at a dose of 60 Gy as previously described^8^, which has been confirmed to lead to RN development 6–8 months after the irradiation^7^. The progression of RN (edema or micro-bleeding) was checked once per month by magnetic resonance imaging for small animals at the Radioisotope Research Center, Kyoto University, as previously described^7^. X-ray irradiation was applied to an area of the right hemisphere (10 mm in height, 5 mm in width) at a dose of 40 Gy. We used this X-ray irradiation model as a representative which do not develop RN.

### Sample collection and lipid extraction procedure for liquid chromatography tandem mass spectrometry (LC–MS/MS) analyses

After deep anesthesia using a combination of anesthetic with 0.3 mg/kg medetomidine, 4.0 mg/kg of midazolam, and 5.0 mg/kg of butorphanol by intraperitoneal injection, and perfusion with cold phosphate-buffered saline, brains were collected at 2, 7 days and 1 month for the X-ray irradiation group and at 10 days 1, 2, 4 and 8 months for RN model groups after irradiation. Brains were cut at the center line and by removal of brainstem and cerebellum, the rest part of brains (cerebral cortex and limbic system, etc.) were snap frozen with liquid nitrogen before being stored at − 80 °C until use. Each tissue specimen was placed in a 1.5-mL sample tube, and 1 mL of acidic methanol (pH 4.0) and the internal standards (17:0 LPA, 17:0 LPC, 24:0 PA, 24:0 PS, and 24:0 PC) were added. Zirconium beads were added to the mixture in the tube, and homogenization was performed via sonication for 10 min. The resulting supernatants were harvested in tubes and centrifuged. These extracts were then subjected to LC–MS/MS analyses.

### LC–MS/MS analysis

We performed LC–MS/MS analyses of the samples according to a previously described method^16^. A methanol extract from 10 mL of the lipid sample was obtained using an UltiMate 3000 (Thermo Fisher Scientific, Tokyo, Japan) equipped with a C18 CAPCELL PAK ACR column (1.5 × 250 mm^2^; Shiseido, Kyoto, Japan) for LPLs or a C8 UG120 CAPCELL PAK ACR column (1.5 × 150 mm^2^; Shiseido) for PLs, with a gradient of solvent A (5 mM ammonium formate in water, pH 4.0) and solvent B (5 mM ammonium formate in 95% [volume/volume] acetonitrile, pH 4.0). The MS/MS was conducted using a TSQ Quantiva triple quadrupole mass spectrometer (Thermo Fisher Scientific). LPA, LPE, LPG, LPI, and LPS were monitored in the negative ion mode, whereas LPC and all PLs were monitored in the positive ion mode. The ions produced were as follows: *m*/*z* 153.0 (LPA) and *m*/*z* 184.1 (LPC). For each LPL class, 11 acyl chains (14:0, 16:0, 16:1, 18:0, 18:1, 18:2, 18:3, 20:3, 20:4, 20:5, 22:5, and 22:6) were monitored. For each PL class, 16 acyl chains (32:0, 32:1, 34:0, 34:1, 34:2, 36:1, 36:2, 36:4, 38:3, 38:4, 38:5, 38:6, 40:5, 40:6, and 40:7) were monitored. We calculated the concentrations of LPLs or PLs by obtaining the ratios between the LPL peaks or PL peaks and the IS peaks (100 nM of 17:0 LPA for LPA, LPE, LPI, LPG, and LPS species; 1 µM of 17:0 LPC for LPC species; 1 µM of 24:0 PA for PA, PE, PI, and PG species; 1 µM of 24:0 PS for PS species; and 5 µM of 24:0 PC for PC species). The ratios of peak areas between the analytes and internal standards were then used to quantify the LPLs or PLs in samples using a calibration curve. These ratios were corrected by each sample weight (g). For this analysis, we performed the high-performance liquid chromatography with an elongated elution time.

### IMS sample preparation and matrix-assisted laser desorption/ionization (MALDI)-IMS analyses

The collection of brain samples was performed as described in the LC–MS/MS analysis section. Tissues blocks were sectioned at − 16 °C using a cryostat (CM 1950; Leica, Wetzlar, Germany) at a thickness of 6 µm. The frozen sections were thaw-mounted on indium-tin-oxide-coated glass slides (Bruker Daltonics, Billerica, MA, USA). These slides were used for the tandem time-of-flight (TOF/TOF) measurements. Prepared sections were subjected to matrix application within 5 min of preparation. A dihydroxybenzoic acid (DBH) solution (40 mg/mL DHB, 20 mM potassium acetate, 70% MetOH, and 0.1% trifluoroacetic acid) was used as the matrix solution for the imaging of PCs and LPCs. For the imaging of LPAs, a solution of Phos-tag MS-101H (^68^Zn) (1 mM aqueous solution) dissolved in trihydroxyacetophenone (100 mg/mL in 50% acetonitrile) at a 1:2 ratio was used as the matrix solution. Phos-tag MS-101H (^68^Zn) binds to LPA and forms complexes that can be efficiently detected by MALDI-TOF MS^17^. The matrix solution was sprayed over the tissue surface using a 0.2-mm nozzle caliber airbrush (Procon Boy FWA Platinum; Mr. Hobby, Tokyo, Japan). Matrices were applied simultaneously to tissue sections that were to be compared with equalized analyte extraction and co-crystallization conditions. For PC and LPC detection, MALDI-IMS analyses were performed using a MALDI-Fourier transform ion cyclotron resonance type instrument (Solarix 7.0T; Bruker Daltonics) at the Research Center for Artificial Photosynthesis in Osaka Metropolitan University. The laser was set to the small spot size with 26% laser power. We obtained positive ions in a mass range of *m*/*z* 400 to 1000. The laser scan pitch was set to 100 µm. For LPA detection, MALDI-IMS analyses were performed using a MALDI-Fourier transform ion cyclotron resonance type instrument (Solarix XR; Bruker Daltonics) at Hamamatsu University School of Medicine. The laser was set to the small spot size with 50% laser power. We obtained positive ions in a mass range of *m*/*z* 500 to 1500. The laser scan pitch was set to 25 µm. Calibration of the *m*/*z* values was performed for each MALDI-IMS measurement using a standard calibration substance (sodium formate). Signals were collected using flexControl software (Bruker Daltonics), and reconstruction of the ion images (normalized by total ion current) was performed using flexImaging 4.0 software (Bruker Daltonics).

### Treatment with ivermectin

Ivermectin (Sigma-Aldrich, Tokyo, Japan) was administered to the treated RN mouse group at a concentration of 12 mg/L in their drinking water. This treatment dosage was chosen based on the treatment of an ALS mouse model in a previous study^15^. The non-treated RN mice received the same amount of the solvent (ethanol) in their drinking water. Treatment started 2 days after irradiation and continued thereafter.

### Immunohistochemistry

After deep anesthesia using a combination of anesthetic with 0.3 mg/kg medetomidine, 4.0 mg/kg of midazolam, and 5.0 mg/kg of butorphanol by intraperitoneal injection and perfusion with cold phosphate-buffered saline and 4% paraformaldehyde, brains were collected and stored at 4 °C overnight. The target proteins were stained in frozen sections for Supplementary Figs. 1 and 2 and in formalin-fixed, paraffin-embedded tissue sections using single and double immunohistochemistry for Fig. 4. The standard avidin–biotin complex method was used. Briefly, serial sections were prepared and for formalin-fixed, paraffin-embedded tissue, deparaffinized, and gradual rehydration was performed. The sections were then heated in antigen retrieval buffer (sodium citrate pH 6.0) before being incubated with 0.3% (volume/volume) hydrogen peroxide for 30 min to inactivate any endogenous peroxidase activity. We used 5% normal horse serum (94010, VECTOR, Burlingame, USA)/PBS in the blocking buffer and expose tissues to it for 30 min. Anti-ionized calcium binding adaptor molecule 1 (Iba-1) rabbit polyclonal antibody (1: 400, 019-19741, FUJIFILM Wako, Tokyo, Japan), anti-glial fibrillary acidic protein: (GFAP) mouse monoclonal antibody (1: 250, G3893, Sigma-Aldrich, Tokyo, Japan) and anti-P2RX4 rabbit polyclonal antibody (1:400, APR-002, Alomone Labs, Jerusalem, Israel) were used for the immunohistochemical analyses for 3 h at room temperature. Reactivity with the primary antibody was detected by incubating the sections with biotin-labeled goat anti-mouse/rabbit IgG (1:250, in 0.5% BSA/PBST) for 3 h at room temperature followed by treatment with the avidin–biotin–peroxidase complex (ABC Kit; Vector, Burlingame, CA, USA). The 3,3′-diaminobenzidine tetrahydrochloride (DAB) solution (DAKO, Kyoto, Japan) was used for antigen visualization. All immunohistochemical procedures were optimized by testing negative controls and antigen retrieval methods. For the double immunohistochemistry, the primary antibodies used for Iba-1 were goat (1:200, ab5076, Abcam, Cambridge, UK), and for P2RX4 they were the same as for DAB staining, and incubated for 3 h at room temperature. Iba-1 was visualized with Alexa Fluor 594 anti-goat antibody (1:200, in 0.5% BSA/PBST, Thermo Fisher Scientific) and P2RX4 was visualized with Alexa Fluor 488 anti-rabbit antibody (1:200, in 0.5% BSA/PBST, Thermo Fisher Scientific) with 3 h exposure at room temperature. The sections were then mounted with coverslips using 50% glycerol with 4′,6-diamidino-2-phenylindole (Santa Cruz Biotechnology, Santa Cruz, CA, USA). For Fig. 4, images were acquired using a BZ-X710 microscope (Keyence, Osaka, Japan) and digitally processed, and the numbers and areas of Iba-1^+^ cells and P2RX4^+^ cells, as well as the total areas in cerebral cortex of brain sections, were measured using BZ-X analyzer software (Keyence). For Supplementary Figs. 1 and 2, images were acquired using a microscope (BX-50, Olympus, Tokyo, Japan) with a digital camera (DFC-7000T, Leica Microsystems, Tokyo, Japan). The numbers and areas of Iba-1^+^ cells and GFAP^+^ cells in cerebral cortex or dentate gyrus of brain sections were measured.

### Behavioral analyses for learning and memory

Before all tests, there were no differences in the weights of mice among the non-irradiated control, non-treated RN, and ivermectin-treated RN groups. All tests were performed 5 months after irradiation.

#### Rotarod test

This test was performed using a 30-mm diameter rotarod treadmill (Muromachi Kikai, Tokyo, Japan) to evaluate motor coordination. Mice were initially trained to stay on the rotating rod. In the test trials, the time (s) that trained mice were able to remain on the rod, which rotated at 25 rpm, was recorded.

#### Open field test

Spontaneous locomotor activity was measured in a square arena (Muromachi Kikai) using a device outfitted with photo-beam detectors for monitoring horizontal and vertical activity. The total time traveled, total distance traveled, and total time exploring the central, middle, and outer areas were recorded. Mice were allowed to explore freely for 60 min while data were collected.

#### Y-maze test

Exploratory behavior was tested using the Y-maze test. The Y-maze apparatus (Muromachi Kikai) consisted of a gray plastic wall (12 cm high) making up three compartments (40 × 2 cm) connected by 2 × 2 cm passages. The mice were placed into one of the three arms of the maze and allowed to explore the two open arms for 5 min, during which the third arm remained closed (training trial). After a 1-h interval, the closed arm was opened, and the mice were allowed to explore all three arms for 5 min (test trial). An arm entry was recorded when all four paws entered the compartment. Performance was monitored using the CompACT VAS/DV video-tracking system (Muromachi Kikai).

### Statistical analysis

Data are presented as the mean ± standard error of the mean, and the sample sizes and numbers of repeats are indicated in the figure legends. PL and LPL data were analyzed using one-way analysis of variance. Comparisons between two groups were conducted using the unpaired Student’s two-tailed *t*-test for LPLs and PLs. The immunohistochemical data were analyzed using one-way analysis of variance or the unpaired *t*-test (Graph Pad Prism 9.2.0; GraphPad Software, San Diego, CA, USA). Survival data were analyzed using the log-rank test (Graph Pad Prism 9.2.0). Behavioral data were analyzed using the Bonferroni multiple comparisons test.

### Ethics approval

All animal experiments were performed according to procedures that were approved by the Animal Experiment Committee of Kyoto University (No. 23 in 2017, No. 20 in 2018 and No. 40 in 2019) and the Wakasa Wan Energy Research Center (No. 3001-1, NO. 201801 and No. 201901).

## Results

### Phospholipids (PLs) were elevated at 1 month and gradually decreased, while LPLs were elevated at 1 month and remained high after irradiation

**Figure 1:**
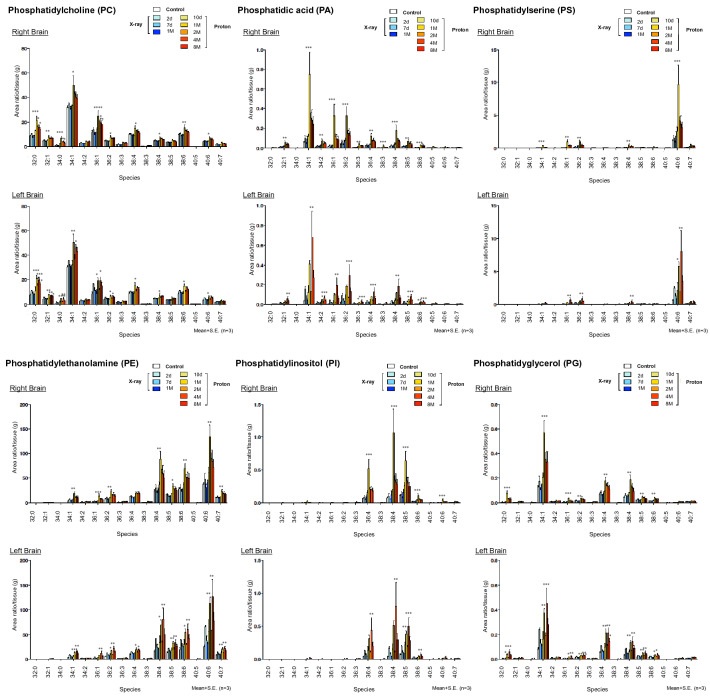
Phospholipid changes after irradiation in the brain radiation necrosis (RN) model as detected by LC–MS/MS. Quantitative changes of phosphatidylcholine (PC), phosphatidyl acid (PA), phosphatidylserine (PS), phosphatidylethanolamine (PE), phosphatidylinositol (PI) and phosphatidylglycerol (PG) of the right or left hemisphere of control or irradiated mice at 10 days, 1 month, 2 months, 4 months, and 8 months after proton beam irradiation, or at 2 days, 7 days, and 1 month after X-ray irradiation (*n* = 3 per group). Upper graphs indicate the right hemisphere and lower graphs indicate the left hemisphere. *S. E.* standard error. *P < 0.05, **P < 0.01, ***P < 0.001, ****P < 0.0001 versus non-irradiated control mice; one-way analysis of variance.

**Figure 2:**
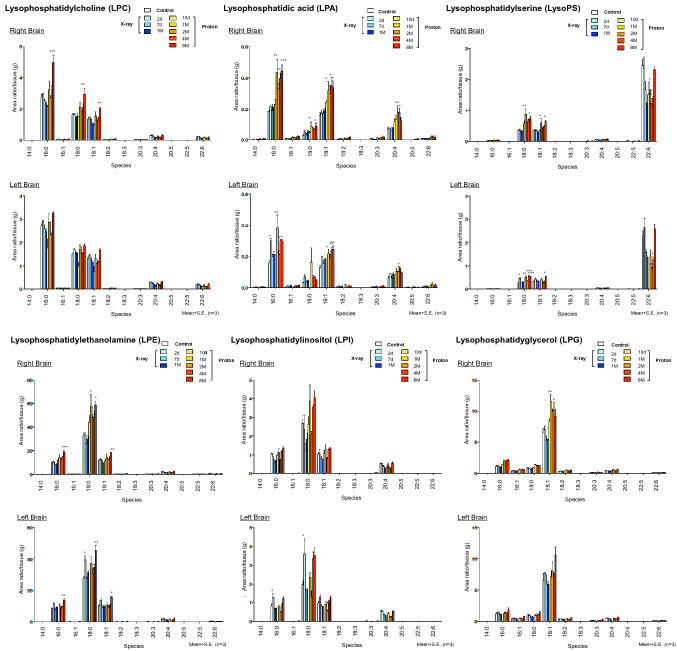
Lysophospholipid changes after irradiation in the brain RN model as detected by LC–MS/MS. Quantitative changes of lysophosphatidylcholine (LPC), lysophosphatidyl acid (LPA), lysophosphatidylserine (LPS), lysophosphatidylethanolamine (LPE), lysophosphatidylinositol (LPI), and lysophosphatidylglycerol (LPG) of the right or left hemisphere of control or irradiated mice at 10 days, 1 month, 2 months, 4 months, or 8 months after proton beam irradiation, or at 2 days, 7 days, and 1 month after X-ray irradiation (*n* = 3 per group). Upper graphs indicate the right hemisphere and lower graphs indicate the left hemisphere. *S. E.* standard error. *P < 0.05, **P < 0.01, ***P < 0.001 versus non-irradiated control mice; one-way analysis of variance.

**Figure 3:**
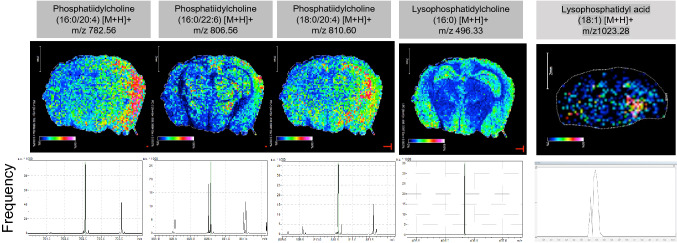
Images of PC, LPC and LPA in the brain RN model 1 month after irradiation. Schema of a mouse brain coronal section and ion images of phosphatidylcholines (PCs), lysophosphatidylcholine (LPC), and lysophosphatidyl acid (LPA) obtained by IMS are shown. Distributions of PCs (16:0/20:4), (16:0/22:6), and (18:0/20:4), LPC (16:0), and LPA (18:1) in brain sections of the RN model 1 month after irradiation are indicated. All data were normalized using total ion currents. A representative mass spectrum obtained from an RN mouse brain section using matrix-assisted laser desorption/ionization (MALDI)-IMS is shown for each PC, LPC, and LPA.

In the irradiated right hemisphere of the RN model, all PLs (PC, phosphatidyl acid [PA], PS, PE, phosphatidylinositol [PI], and phosphatidylglycerol [PG]) reached their peaks at 1 month and were significantly increased compared with controls; they then gradually decreased from 2 to 8 months after irradiation. In the non-irradiated left hemisphere, PC, PA, PS, PE, PI, and PG were significantly increased at 1 month, decreased at 2 months, significantly increased again at 4 months, and then gradually decreased (Fig. 1). In contrast, LPC reached its peak at 8 months and was significantly increased compared with controls. LPA, lysophosphatidylserine (LysoPS), lysophosphatidylethanolamine (LPE), and lysophosphatidylglycerol (LPG) were significantly increased at 1 month, and then re-elevated and remained significantly higher compared with controls until 4 or 8 months in both the irradiated and non-irradiated areas (Fig. 2). X-ray irradiation to the right hemisphere at a dose of 40 Gy, which does not cause RN, did not cause as large an increase in PLs or LPLs as proton beam irradiation, and the increase was not sustained in either PLs or LPLs. Furthermore, in the non-irradiated left hemisphere, LPA, LysoPS, LPE, and LPI were significantly increased at 2 days compared with controls after X-ray irradiation. The distributions of PCs (16:0/20:4), (16:0/22:6), (18:0/20:4), LPC (16:0), and LPA (18:1) appeared relatively elevated in the irradiated area of the RN model 1 month after irradiation using imaging mass spectrometry (IMS) analysis (Fig. 3). LPA (18:1) was detected as a [^68^Zn] Phos-tag complex.

### Ivermectin decreased the accumulation of microglia and P2RX4 in the RN model

**Figure 4:**
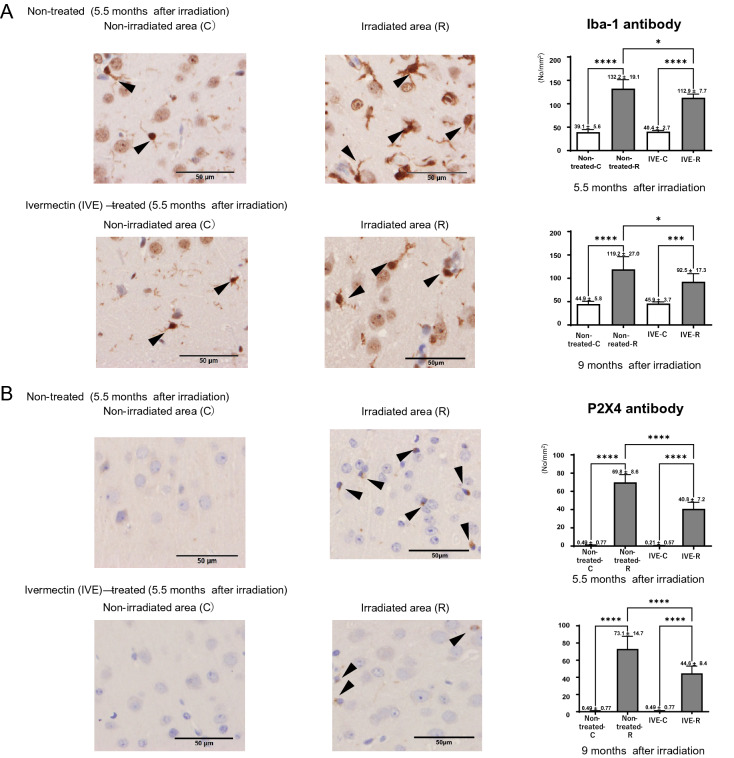

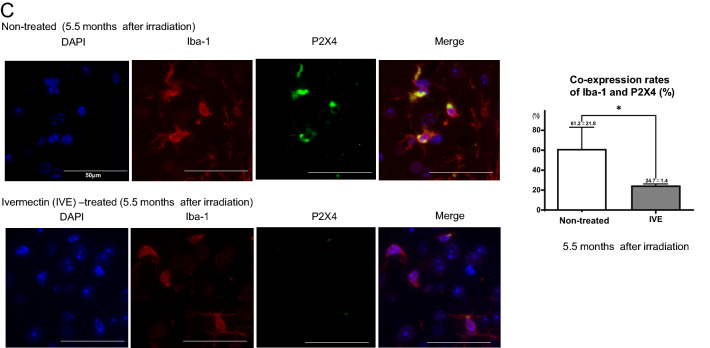
Immunostaining of Iba-1 and P2RX4 between non-treated and Ivermectin (IVE)-treated RN model mice. Histology of the non-irradiated (C) and irradiated (R) areas using (**A**) Iba-1 antibodies and (**B**) P2RX4 antibodies in the non-treated and IVE-treated brain radiation necrosis mice at 5.5 months after irradiation. Bar graphs indicate the numbers in each group (non-treated: *n* = 6 and IVE-treated: *n* = 7). The same is shown for mice at 9 months after irradiation (non-treated: *n* = 6 and IVE-treated: *n* = 6). Scale bar = 50 µm. ***P < 0.001, ****P < 0.0001 versus non-irradiated mice, *P < 0.05, ****P < 0.0001 versus non-treated mice, one-way analysis of variance. (**C**) Co-expression of Iba-1 (red) and P2RX4 (green) in the irradiated area at 5.5 months after irradiation. Bar graphs indicate the percentage of co-expression rates (%) (non-treated: *n* = 3 and IVE-treated: *n* = 3). *P < 0.05, unpaired *t*-test.

In preliminary experiments, we confirmed an increase in the number of microglia only in the irradiated area including both cerebral cortex and dentate gyrus with the development of RN (Supplementary Fig. 1A,B). On the other hand, the number of astrocytes showed gradually decrease in the irradiated cerebral cortex and dentate gyrus after 3 months irradiation (Supplementary Fig. 2A,B). In the present study, in RN mice, the numbers of microglia in cerebral cortex were significantly increased in the irradiated area compared with the non-irradiated area in both the non-treated and ivermectin-treated groups at 5.5 months and 9 months after irradiation. Ivermectin treatment significantly reduced the numbers of microglia (Iba-1^+^ cells) in the irradiated area at 5.5 months (non-treated: 132.2 ± 19.1, ivermectin-treated: 112.9 ± 7.7) and 9 months (non-treated: 119.2 ± 27, ivermectin-treated: 92.5 ± 17.3) after irradiation (Fig. 4A). P2RX4 expression was detected in the irradiated area of cerebral cortex, only in both the non-treated and ivermectin-treated groups. Ivermectin treatment significantly decreased the numbers of P2RX4^+^ cells from 69.8 ± 8.6 to 40.8 ± 7.2 at 5.5 months, and from 73.1 ± 14.7 to 44.6 ± 8.4 at 9 months (Fig. 4B). The co-expression rate (%) of P2RX4 and Iba-1 in cerebral cortex was significantly lower in the ivermectin-treated group (24.7%) than in the non-treated group (61.3%) at 5.5 months after irradiation (Fig. 4C).

### Ivermectin decreased PCs and LPLs, except for LPC, in the RN model

**Figure 5:**
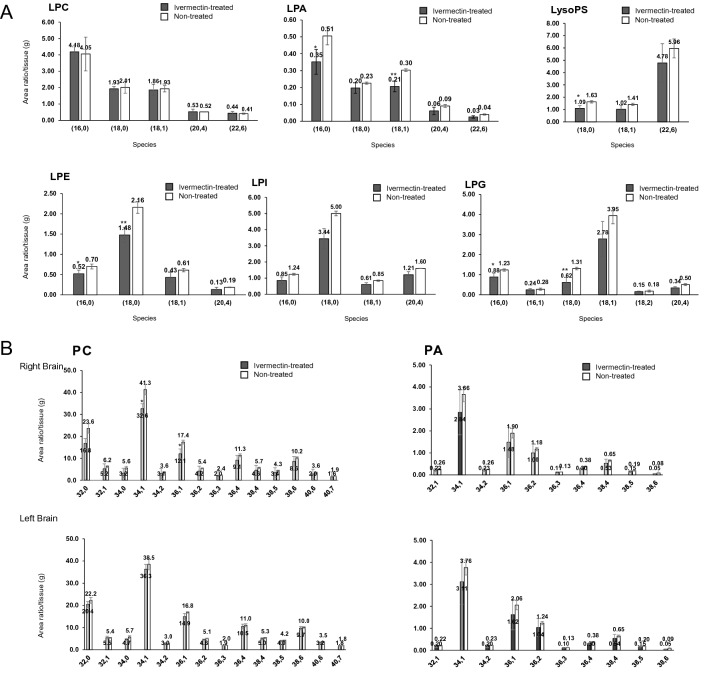
Quantitative change of LPLs, PC and PA in the brain RN mice by IVE-treatment. (**A**) Quantitative comparison of lysophosphatidylcholine (LPC), lysophosphatidyl acid (LPA), lysophosphatidylserine (LPS), lysophosphatidylethanolamine (LPE), lysophosphatidylinositol (LPI), and lysophosphatidylglycerol (LPG) between non-treated and IVE-treated mice (*n* = 3 per group) in the irradiated area at 5.5 months after irradiation in the brain radiation necrosis (RN) model. (**B**) Quantitative comparison of phosphatidylcholine (PC) and phosphatidyl acid (PA) between non-treated and IVE-treated mice (*n* = 3 per group) in the irradiated area at 5.5 months after irradiation in the RN model. Upper graphs indicate the right hemisphere and lower graphs indicate the left hemisphere (non-treated: *n* = 3 and IVE-treated: *n* = 3). *P < 0.05, **P < 0.01, unpaired *t-*test.

At 5.5 months after irradiation, ivermectin treatment reduced the amount of LPA, LPE, LysoPS, LPI, and LPG in the irradiated area to 60–70% of that in the non-treated group (Fig. 5A). Significant reductions were detected in (16:0) LPA (ivermectin-treated: 0.35 ± 0.07 vs non-treated: 0.51 ± 0.05), (18:1) LPA (ivermectin-treated: 0.21 ± 0.03 vs non-treated: 0.30 ± 0.008), (18:0) LysoPS (ivermectin-treated: 1.09 ± 0.22 vs non-treated: 1.63 ± 0.09), (16:0) LPE (ivermectin-treated: 0.52 ± 0.08 vs non-treated: 0.70 ± 0.06), (16:0) LPG (ivermectin-treated: 0.88 ± 0.19 vs non-treated: 1.23 ± 0.06), (18:0) LPG (ivermectin-treated: 0.62 ± 0.25 vs non-treated: 1.31 ± 0.06). Among the LPLs, only LPC was not decreased by ivermectin. Although most PA in both the irradiated and non-irradiated areas had a trend toward a 70–80% decrease with ivermectin treatment compared with the non-treated group, this reduction was not significant (Fig. 5B). However, among most PCs, which are the precursor to LPCs, ivermectin treatment led to a greater reduction in the irradiated area than in the non-irradiated area (Fig. 5B). PC (34:1, 36:1) was significantly reduced in the ivermectin-treated group compared with the non-treated group ((34:1) PC, ivermectin-treated: 32.6 ± 4.8 vs non-treated: 41.3 ± 2.3, (36:1) PC, ivermectin-treated: 12.1 ± 3.0 vs non-treated: 17.4 ± 0.8).

### Ivermectin did not significantly affect survival time or working memory in the RN model

**Figure 6:**
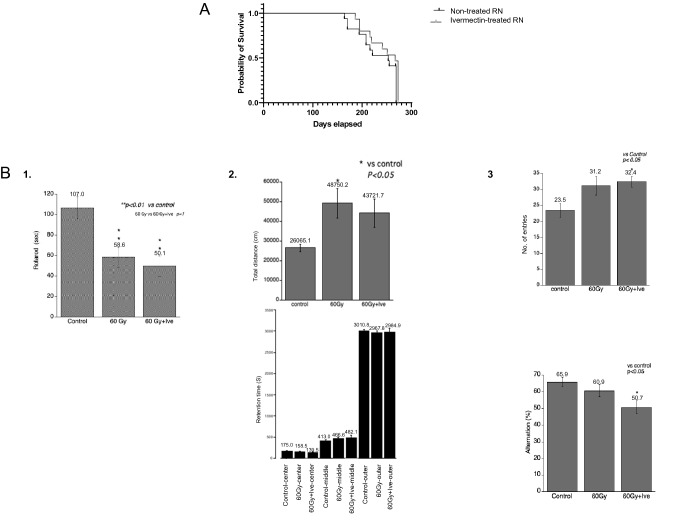
Survival and behavior analyses of the IVE-treated or non-treated brain RN model. (**A**) There was a slight, but non-significant, improvement in survival time in IVE-treated RN mice (*n* = 15) compared with non-treated RN mice (*n* = 17). (**B**) Results from cognitive tests among the non-irradiated control, non-treated RN, and IVE-treated RN groups (*n* = 12 per group). 1. Rotarod test (25 rpm/min). 2. Open field test. 3. Y-maze test. **P < 0.01, *P < 0.05 versus the non-irradiated control group, Bonferroni test.

Although ivermectin did not significantly prolong survival after irradiation, there was a trend for the treated group to live slightly longer than the non-treated group (Fig. 6A). The median survival times for non-treated and ivermectin-treated RN mice were 253 and 267 days, respectively. Among the survivors, RN occurred in two mice in the non-treated group (one was accompanied by hemorrhage) and in no mice in the ivermectin-treated group (*n* = 6 per group).

At 5 months after irradiation, behavioral analyses were performed to investigate the effects of ivermectin on learning and memory in the RN model. In the rotarod test (25 rpm/min), RN model mice remained on the rod for a shorter time compared with the non-irradiated control mice, and ivermectin treatment did not improve the time (Fig. 6B-1). In the open field test, the total distance traveled in the non-treated RN group was twice that of the non-irradiated control group; with ivermectin treatment, there was a trend toward a decrease in the total distance. The time spent in the center, middle, or outer area was not different between the control, non-treated RN, and ivermectin-treated RN groups (Fig. 6B-2). In the Y-maze test, the number of entries was increased in both the non-treated and ivermectin-treated RN groups compared with the non-irradiated control group. In contrast, alteration (%) was decreased in the non-treated and ivermectin-treated RN groups compared with the control group (Fig. 6B-3).

## Discussion

It has recently been noted that LPLs at elevated levels (relative to physiological concentrations) can convert themselves to conditional danger-associated molecular patterns (DAMPs) and initiate sterile inflammation in coronary artery disease^18^ and atherosclerosis^19^. Most LPLs, such as LPA, sphingosine-1-phosphate, LPC, LPG, and LPI, are increasingly assumed to represent disease markers; two exceptions are LysoPS and LPE, which seem to exert anti-inflammatory effects^20^. LysoPS has roles in the promotion of phagocytosis of apoptotic cells and the resolution of inflammation^21^. The anti-inflammatory action of LPE in zymosan A-induced peritonitis has also been reported^22^. From these findings, LysoPS and LPE are now recognized as homeostasis-associated molecular patterns (HAMPs)^20^. Regarding DAMPs, LPA signaling is linked to brain injury, ischemia-induced hypoxia, Alzheimer’s disease, nerve injury, pain, and brain tumors, and concentrations in the brain and cerebrospinal fluid are elevated in these conditions^23^. As well as in brain disorders, LPA is significantly elevated in systemic pathophysiological statuses, such as angiogenesis, inflammation, atherosclerosis, insulin resistance, and obesity^24^. LPC can also accumulate in pathological conditions, such as ischemia and atherosclerotic aortas, and alters a range of functions in many different cell types, including endothelial cells, smooth muscle cells, monocytes, macrophages, microglia, T cells, and B cells^20^.

In our RN model, which was accompanied by chronic inflammation, the investigated DAMPs (LPC, LPA, LPI, and LPG) and HAMPs (LysoPS and LPE) remained at high levels from 1 month after irradiation. These results suggest that RN develops its pathogenesis along with the elevation of LPLs—including both DAMPs and HAMPs—for tissue homeostasis under sterile chronic inflammation. PLs gradually decreased after 1 month but remained at higher levels than in non-irradiated controls; this higher PLs can be the source of LPL production by hydrolysis^2^. PL biosynthesis increases in the endoplasmic reticulum (ER) under stress to increase the surface area and volume of rough ER, and there is enhanced activity of the cytidine diphosphocholine pathway of phosphatidylcholine biosynthesis^25^. Therefore, the increase in PLs may indicate ER stress caused by chronic inflammation, as reported in Ref.^26^, in the RN model. Among PLs, PS is known to have strong correlations with cognitive function^27^ and reduces the peroxidation of lipids^28^. A transient elevation of PS may therefore indicate its role in protecting brain function from proton irradiation-induced peroxidation or following apoptosis^29^.

A long-lasting elevation of LPLs, and especially LPC and LPA, should lead to the recruitment of microglia in the RN model because these LPLs are well known chemoattractants for immune cells and microglia^20^. Microglial number in irradiated area continued to increase in RN model with time course (Supplementary Fig. 1A,B), while astrocyte numbers in irradiated cerebral cortex of RN model, increased three months after irradiation but began to decrease thereafter (Supplementary Fig. 2A). Therefore, we considered the persistent LPLs elevation might correlate well with persistent increase in numbers of microglia rather than astrocytes, and further promoted analysis on microglia and P2RX4 expression in RN model. Astrocytes also express P2RX4 at low level, but there is no functional evidence using electrophysiology, of the presence of P2RX4 in hippocampal astrocytes^30^. P2RX4 is mainly expressed in microglial cells and P2RX4^+^ state of microglia seems to be associated to the microglial activation occurring in different paradigms of neuroinflammation^30^.

In the present study, ivermectin treatment decreased the numbers of microglia and P2RX4^+^ cells in the RN model. In the acute stage of ischemic stroke in the human brain, in the zone surrounding the fresh infarct (known as the penumbra), microglia are activated by LPC derived from adjacent neurons and astrocytes through G2A or P2RX7 to produce the inflammatory chemokines monocyte chemoattractant protein-1 (MCP-1) and C–C chemokine receptor type 2 (CCR2)^11^. Together, these results suggest that the blockade of these receptors may be a promising therapeutic strategy for suppressing the proinflammatory response of microglia in hypoxic stress-induced inflammation. However, in EAE (an animal model of multiple sclerosis, which is another chronic inflammatory disease of the brain and spinal cord), the blockade of P2RX4 signaling exacerbates clinical signs, favors microglial activation to a pro-inflammatory phenotype, and inhibits myelin phagocytosis. Moreover, P2RX4 blockade in microglia halts both oligodendrocyte differentiation in vitro and remyelination after LPC-induced demyelination. Conversely, the potentiation of P2RX4 signaling by ivermectin favors a switch in microglia to an anti-inflammatory phenotype, potentiates myelin phagocytosis, promotes the remyelination response, and ameliorates the clinical signs of EAE^14^. Ivermectin also protects against hypoxia/hypoglycemia-induced motor neuron death^15^. ALS is a neurodegenerative disease that is caused by the degeneration of motor neurons. Ivermectin treatment of SOD1-G93A mice (a transgenic animal model of familial ALS) has been reported to extend the lifespan of these mice by almost 10%^15^. Based on these reports, we decided to use ivermectin for RN treatment in the present study, and revealed the effectiveness of ivermectin for repressing the recruitment of microglia and their activation through P2RX4. This repression by ivermectin may have been caused by reduced LPA and persistently high LPC (even with ivermectin treatment), which may be associated with the limited amount of microglial reduction.

It remains unclear why ivermectin did not reduce LPC, although other LPLs were reduced by this treatment. As a result, chronic inflammation was not completely blocked under persistently high LPC, which is one of the major DAMPs in chronic inflammation besides reductions of LysoPS and LPE which are known as HAMPs to exert anti-inflammatory effects^20^. In addition, because PC (the precursor of LPC) was reduced by ivermectin in the irradiated area, persistently high LPC was presumably produced by continuously activated secretory PLA2 (sPLA2), which hydrolyzes PC. The trend toward a slightly prolonged survival with ivermectin treatment in RN may be the result of anti-inflammatory effects, with downregulated LPA, LPI, or LPG. However, the very limited effect on survival was likely caused by persistently high LPC. Furthermore, an ivermectin-induced improvement in short-term memory decline in RN was not achieved or even worsened; again, this was likely because of persistently high LPC, which is a demyelinating agent^31^. LPC can contribute to chronic inflammation through the activation of caspase-1-mediated release of interleukin 1 (IL-1) via stimulation of the Nod-like receptor family pyrin domain containing 3 (NLRP3) and Nod-like receptor family CARD domain containing 4 (NLRC4) inflammasomes^32,33^, and is now recognized as one of the major DAMPs. LPC also triggers the release of another DAMP, ATP, from macrophages and neuronal cells, and reduces the threshold of one or more P2RX to this DAMP to generate cell death and the release of caspase-1-dependent IL-1 beta^34^. Although ivermectin reduced P2RX4 activation in the RN model in the current study, the reduced threshold of P2RX4 induced by LPC, and the subsequent ATP, may be sufficient to cause continuous inflammation and neuronal cell death in RN, even with ivermectin treatment. This extracellular ATP may also promote the production of IL-12p70 from Toll-like receptor-sensitized macrophages; reduce the phagocytic ability of macrophages; and reduce the surface expression of major histocompatibility complex class II, cluster of differentiation 86 (CD86), and several other molecules on these cells. All these responses are abrogated in the presence of the ATP-hydrolyzing enzyme apyrase^34^. Therefore, apyrase may be a promising candidate for blocking LPC-induced chronic inflammation in RN. In addition, sPLA2, the synthetase of LPC, is elevated in multiple brain diseases including Alzheimer’s disease, cerebrovascular disease, multiple sclerosis, spinal cord injury, and epilepsy^35^. It has been reported that infarct volume is significantly reduced in rats treated with the sPLA2 inhibitor 7,7-dimethyleicosadienoic acid^36^. Thus, in RN, treatment with sPLA2 inhibitors can also be considered as a candidate therapeutic strategy.

LPLs stimulate the production of reactive oxygen species (ROS) and trigger oxidative injury in many types of cells. ROS generated by reduced nicotinamide adenine dinucleotide (NADH)/nicotinamide adenine dinucleotide phosphate (NADPH) oxidase contribute to the LPC-induced activation of extracellular signal-regulated kinase 1/2 (ERK1/2) and subsequent growth promotion in vascular smooth muscle cells^37^. Furthermore, LPC-induced ROS generation can be blocked by the NADH/NADPH oxidase inhibitor diphenylene iodonium. Additionally, the antioxidants *N-*acetyl-l-cysteine, glutathione monoester, and α-tocopherol inhibit LPC-induced ERK1/2 activation^37^. Vascular dilatation is also involved in the pathogenesis of RN; therefore, the blockade of these LPC-induced oxidative injuries by diphenylene iodonium or antioxidants may offer advantages as RN treatments.

## Conclusion

In conclusion, the present study revealed that brain RN developed along with the persistent elevation of various LPLs, which was accompanied by microglial activation. This LPL elevation and microglial activation was controlled by treatment with ivermectin, an allosteric modulator of P2RX4, except in the case of LPC, which remained elevated. To achieve further benefits in RN, including prolonged survival and the amelioration of working memory impairment, it may be crucial to block the production of LPC using sPLA2 inhibitors and to block consequent oxidative injury using NADH/NADPH oxidase inhibitors and antioxidants from the early stage, when LPC and other LPLs begin to elevate.

## Supplementary Information

Supplementary Figures.

## Data Availability

All data generated or analysed during this study are included in this published article.

## Abbreviations

ALS: Amyotrophic lateral sclerosis
CCR2: C–C chemokine receptor type 2
CD86: Cluster of differentiation 86
DAMPs: Danger-associated molecular patterns
EAE: Experimental autoimmune encephalomyelitis
ER: Endoplasmic reticulum
ERK1/2: Extracellular signal-regulated kinase 1/2
G2A: G protein-coupled receptor 132
HAMPs: Homeostasis-associated molecular patterns
IL-1: Interleukin 1
IMS: Imaging mass spectrometry
LC–MS/MS: Liquid chromatography tandem mass spectrometry
LPA: Lysophosphatidyl acid
LPC: Lysophosphatidylcholine
LPE: Lysophosphatidylethanolamine
LPG: Lysophosphatidylglycerol
LPLs: Lysophospholipids
LysoPS: Lysophosphatidylserine
MALDI: Matrix-assisted laser desorption/ionization
MCP-1: Monocyte chemoattractant protein-1
NADH: Nicotinamide adenine dinucleotide
NADPH: Nicotinamide adenine dinucleotide phosphate
NLRP3: Nod-like receptor family pyrin domain containing 3
NLRC4: Nod-like receptor family CARD domain containing 4
PA: Phosphatidyl acid
PC: Phosphatidylcholine
PG: Phosphatidylglycerol
PE: Phosphatidylethanolamine
PI: Phosphatidylinositol
P2RX4: Purinergic receptor P2X purinoceptor 4
P2RX7: P2X purinoceptor 7
PLA_2_: Phospholipase A_2_
PS: Phosphatidylserine
RN: Brain radiation necrosis
ROS: Reactive oxygen species
sPLA2: Secretory PLA2

## References

[1] Makale MT, McDonald CR, Hattangadi-Gluth JA, Kesari S (2017). Mechanisms of radiotherapy-associated cognitive disability in patients with brain tumours. Nat. Rev. Neurol..

[2] Zhuang H, Siyu S, Yuan Z, Chang JY (2019). Bevacizumab treatment for radiation brain necrosis: Mechanism, efficacy and issues. Mol. Cancer.

[3] Makide K, Kitamura H, Sato Y, Okutani M, Aoki J (2009). Emerging lysophospholipid mediators, lysophosphatidylserine, lysophosphatidylthreonine, lysophosphatidylethanolamine and lysophosphatidylglycerol. Prostagland. Other Lipid Mediat..

[4] Wang C, Yang J, Nie J (2009). Plasma phospholipid metabolic profiling and biomarkers of rats following radiation exposure based on liquid chromatography-mass spectrometry technique. Biomed. Chromatogr..

[5] Zhao H, Xi C, Tian M, Lu X, Cai TJ, Li S, Tian XL, Gao L, Liu HX, Liu KH (2020). Identification of potential radiation responsive metabolic biomarkers in plasma of rats exposed to different doses of cobalt-60 gamma rays. Dose Response.

[6] Upadhyay M, Rajagopal M, Gill K, Li Y, Bansal S, Sridharan V, Tyburski JB, Boerma M, Cheema AK (2020). Identification of plasma lipidome changes associated with low dose space-type radiation exposure in a murine model. Metabolites.

[7] Kondo N, Sakurai Y, Takata T, Takai N, Nakagawa Y, Tanaka H, Watanabe T, Kume K, Toho T, Miyatake S (2015). Localized radiation necrosis model in mouse brain using proton ion beams. Appl. Radiat. Isot..

[8] Takata T, Kondo N, Sakurai Y, Tanaka H, Hasegawa T, Kume K, Suzuki M (2015). Localized dose delivering by ion beam irradiation for experimental trial of establishing brain necrosis model. Appl. Radiat. Isot..

[9] Ueda H, Matsunaga H, Olaposi OI, Nagai J (2013). Lysophosphatidic acid: Chemical signature of neuropathic pain. Biochem. Biophys. Acta.

[10] Heneka MT, Carson MJ, El Khoury J, Landreth GE, Brosseron F, Feinstein DL, Jacobs AH, Wyss-Coray T, Vitorica J, Ransohoff RM (2015). Neuroinflammation in Alzheimer's disease. Lancet Neurol..

[11] Inose Y, Kato Y, Kitagawa K, Uchiyama S, Shibata N (2015). Activated microglia in ischemic stroke penumbra upregulate MCP-1 and CCR2 expression in response to lysophosphatidylcholine derived from adjacent neurons and astrocytes. Neuropathology.

[12] Kabarowski JH, Zhu K, Le LQ, Witte ON, Xu Y (2001). Lysophosphatidylcholine as a ligand for the immunoregutatory receptor G2A. Science.

[13] Takenouchi T, Sato M, Kitani H (2007). Lysophosphatidylcholine potentiates Ca2^+^ influx, pore formation and p44/42 MAP kinase phosphorylation mediated by P2X7 receptor activation in mouse microglial cells. J. Neurochem..

[14] Zabala A, Vazquez-Villoldo N, Rissiek B, Gejo J, Martin A, Palomino A, Perez-Samartín A, Pulagam KR, Lukowiak M (2018). P2X4 receptor controls microglia activation and favors remyelination in autoimmune encephalitis. EMBO Mol. Med..

[15] Andries M, Van Damme P, Robberecht W, Van Den Bosch L (2007). Ivermectin inhibits AMPA receptor-mediated excitotoxicity in cultured motor neurons and extends the life span of a transgenic mouse model of amyotrophic lateral sclerosis. Neurobiol. Dis..

[16] Kitamura C, Sonoda H, Nozawa H, Kano K, Emoto S, Murono K, Kaneko M, Hiyoshi M, Sasaki K, Nishikawa T (2019). The component changes of lysophospholipid mediators in colorectal cancer. Tumour Biol..

[17] Morishige J, Urikura M, Takagi H, Hirano K, Koike T, Tanaka T, Satouchi K (2010). A clean-up technology for the simultaneous determination of lysophosphatidic acid and sphingosine-1-phosphate by matrix-assisted laser desorption/ionization time-of-flight mass spectrometry using a phosphate-capture molecule, Phos-tag. Rapid Commun. Mass Spectrom..

[18] Kurano M, Suzuki A, Inoue A, Tokuhara Y, Kano K, Matsumoto H, Igarashi K, Ohkawa R, Nakamura K, Dohi T (2015). Possible involvement of minor lysophospholipids in the increase in plasma lysophosphatidic acid in acute coronary syndrome. Arterioscler. Thromb. Vasc. Biol..

[19] Li X, Fang P, Li Y, Kuo YM, Andrews AJ, Nanayakkara G, Johnson C, Fu H, Shan H, Du F (2016). Mitochondrial reactive oxygen species mediate lysophosphatidylcholine-induced endothelial cell activation. Arterioscler. Thromb. Vasc. Biol..

[20] Shao Y, Nanayakkara G, Cheng J, Cueto R, Yang WY, Park JY, Wang H, Yang X (2018). Lysophospholipids and their receptors serve as conditional DAMPs and DAMP receptors in tissue oxidative and inflammatory injury. Antioxid. Redox Signal..

[21] Frasch SC, Bratton DL (2012). Emerging roles for lysophosphatidylserine in resolution of inflammation. Prog. Lipid Res..

[22] Hung ND, Kim MR, Sok DE (2011). 2-Polyunsaturated acyl lysophosphatidylethanolamine attenuates inflammatory response in zymosan A-induced peritonitis in mice. Lipids.

[23] Yung YC, Stoddard NC, Mirendil H, Chun J (2015). Lysophosphatidic acid signaling in the nervous system. Neuron.

[24] Choi JW, Herr DR, Noguchi K, Yung YC, Lee CW, Mutoh T, Lin ME, Teo ST, Park KE, Mosley AN (2010). LPA receptors: Subtypes and biological actions. Annu. Rev. Pharmacol. Toxicol..

[25] Bommiasamy H, Back SH, Fagone P, Lee K, Meshinchi S, Vink E, Sriburi R, Frank M, Jackowski S, Kaufman RJ (2009). ATF6alpha induces XBP1-independent expansion of the endoplasmic reticulum. J. Cell Sci..

[26] Li W, Cao T, Luo C, Cai J, Zhou X, Xiao X, Liu S (2020). Crosstalk between ER stress, NLRP3 inflammasome, and inflammation. Appl. Microbiol. Biotechnol..

[27] Parker AG, Gordon J, Thornton A, Byars A, Lubker J, Bartlett M, Byrd M, Oliver J, Simbo S, Rasmussen C (2011). The effects of IQPLUS focus on cognitive function, mood and endocrine response before and following acute exercise. J. Int. Soc. Sports Nutr..

[28] Yoshida K, Terao J, Suzuki T, Takama K (1991). Inhibitory effect of phosphatidylserine on iron-dependent lipid peroxidation. Biochem. Biophys. Res. Commun..

[29] Tyurina YY, Tyurin VA, Kapralova VI, Wasserloos K, Mosher M, Epperly MW, Greenberger JS, Pitt BR, Kagan VE (2011). Oxidative lipidomics of γ-radiation-induced lung injury: Mass spectrometric characterization of cardiolipin and phosphatidylserine peroxidation. Radiat. Res..

[30] Montilla A, Mata GP, Matute C, Domercq M (2020). Contribution of P2X4 receptors to CNS function and pathophysiology. Int. J. Mol. Sci..

[31] Hall SM (1972). The effect of injections of lysophosphatidyl choline into white matter of the adult mouse spinal cord. J. Cell Sci..

[32] Freeman L, Guo H, David CN, Brickey WJ, Jha S, Ting JP (2017). NLR members NLRC4 and NLRP3 mediate sterile inflammasome activation in microglia and astrocytes. J. Exp. Med..

[33] Scholz H, Eder C (2017). Lysophosphatidylcholine activates caspase-1 in microglia via a novel pathway involving two inflammasomes. J. Neuroimmunol..

[34] Ismaeel S, Qadri A (2021). ATP release drives inflammation with lysophosphatidylcholine. ImmunoHorizons.

[35] Yagami T, Yamamoto Y, Koma H (2014). The role of secretory phospholipaseA2 in the central nervous system and neurological diseases. Mol. Neurobiol..

[36] Hoda MN, Singh I, Singh AK, Khan M (2009). Reduction of lipoxidative load by secretory phospholipase A2 inhibition protects against neurovascular injury following experimental stroke in rat. J. Neuroinflamm..

[37] Yamakawa T, Tanaka S, Yamakawa Y, Kamei J, Numaguchi K, Motley ED, Inagami T, Eguchi S (2002). Lysophosphatidylcholine activates extracellular signal-regulated kinases 1/2 through reactive oxygen species in rat vascular smooth muscle cells. Arterioscler. Thromb. Vasc. Biol..

