# Supplementary material for: Persistent elevation of lysophosphatidylcholine promotes radiation brain necrosis with microglial recruitment by P2RX4 activation
Source: Sci Rep. 2022 May 24;12:8718. doi: 10.1038/s41598-022-12293-3 (PMC9130232; doi:10.1038/s41598-022-12293-3)
Supplement: Supplementary file 1 — Supplementary Figures. [file 41598_2022_12293_MOESM1_ESM.pdf]

# Supplementary figure. 1A

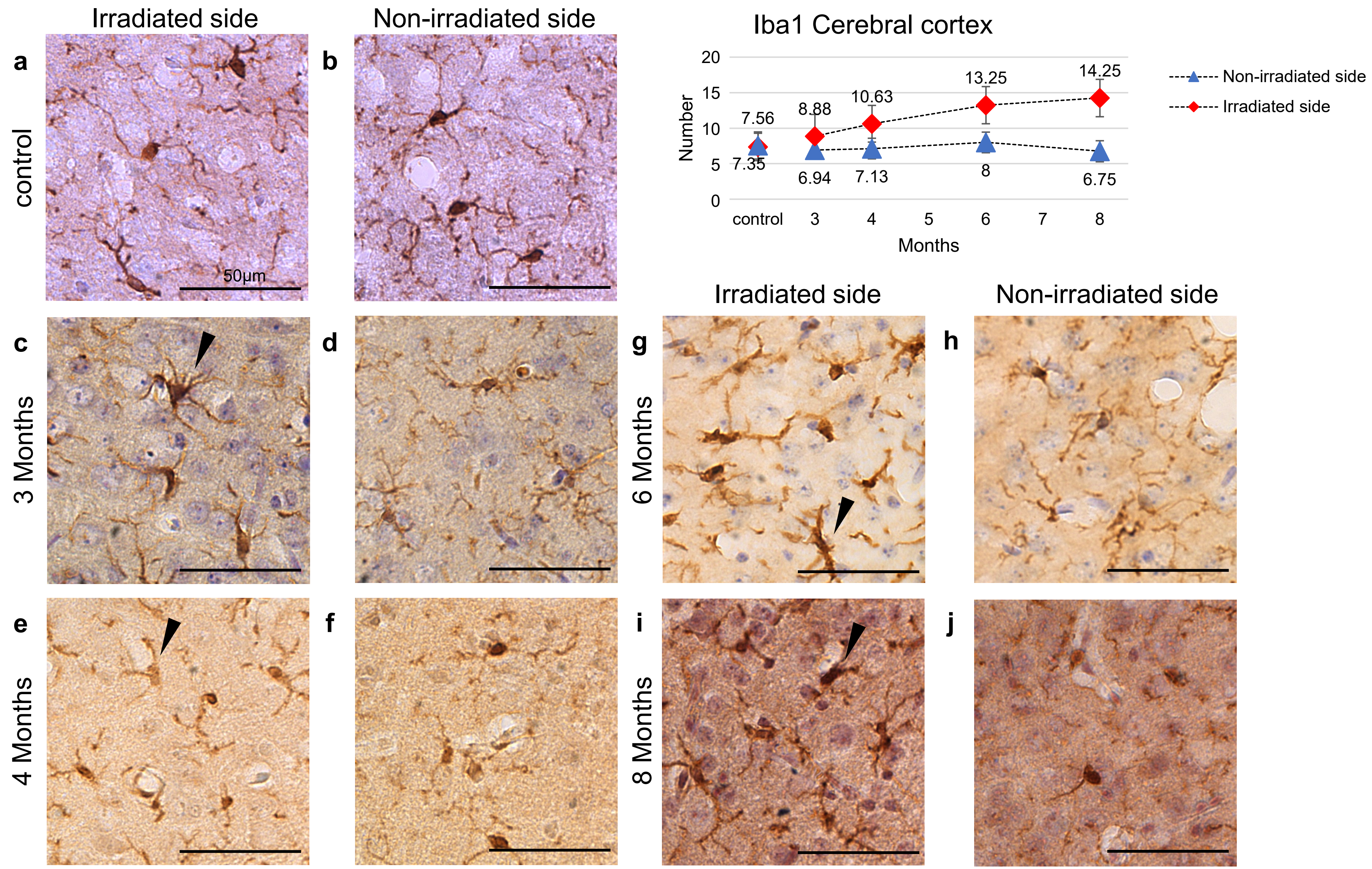

# Supplementary figure. 1B

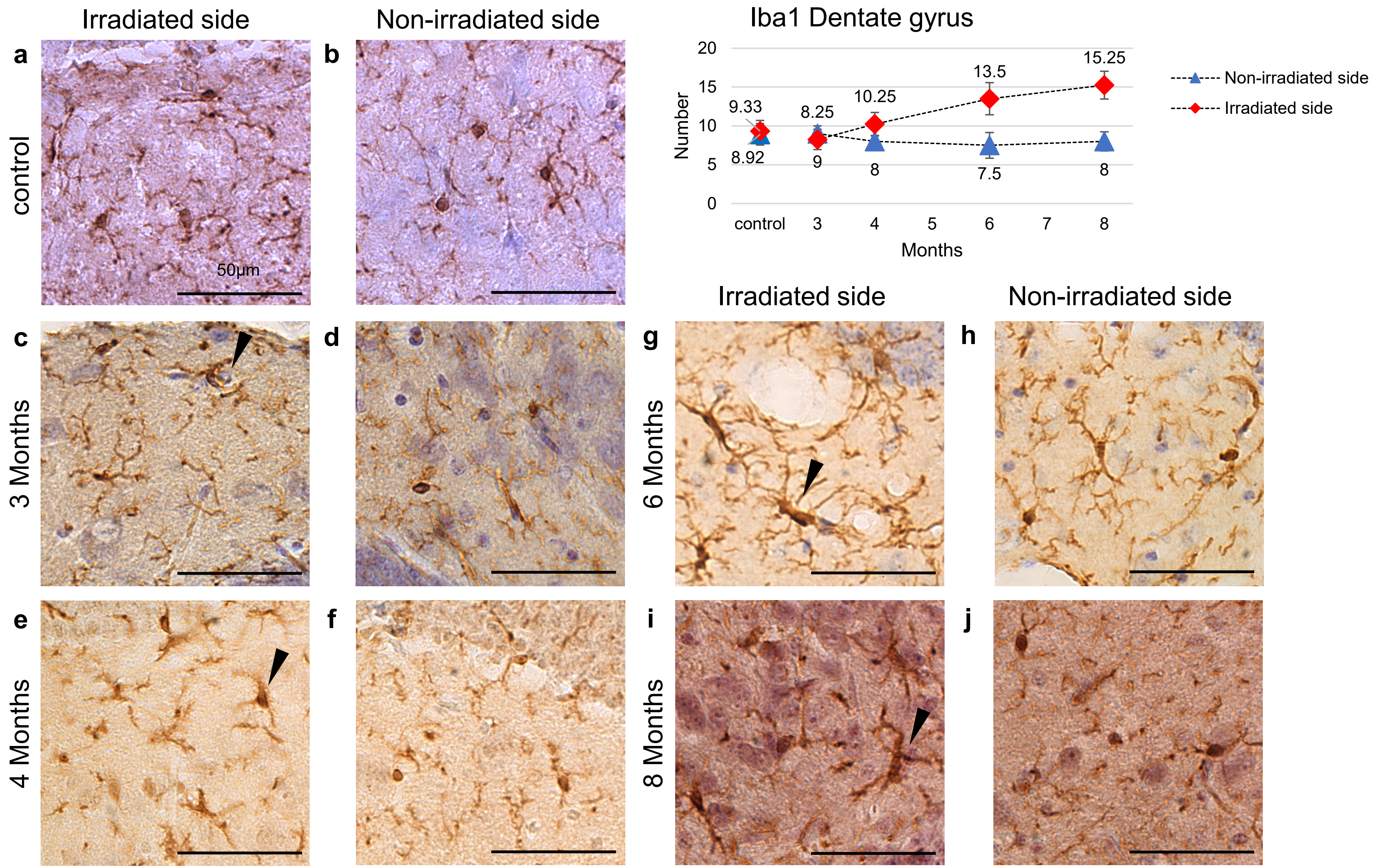

# Supplementary figure. 2A

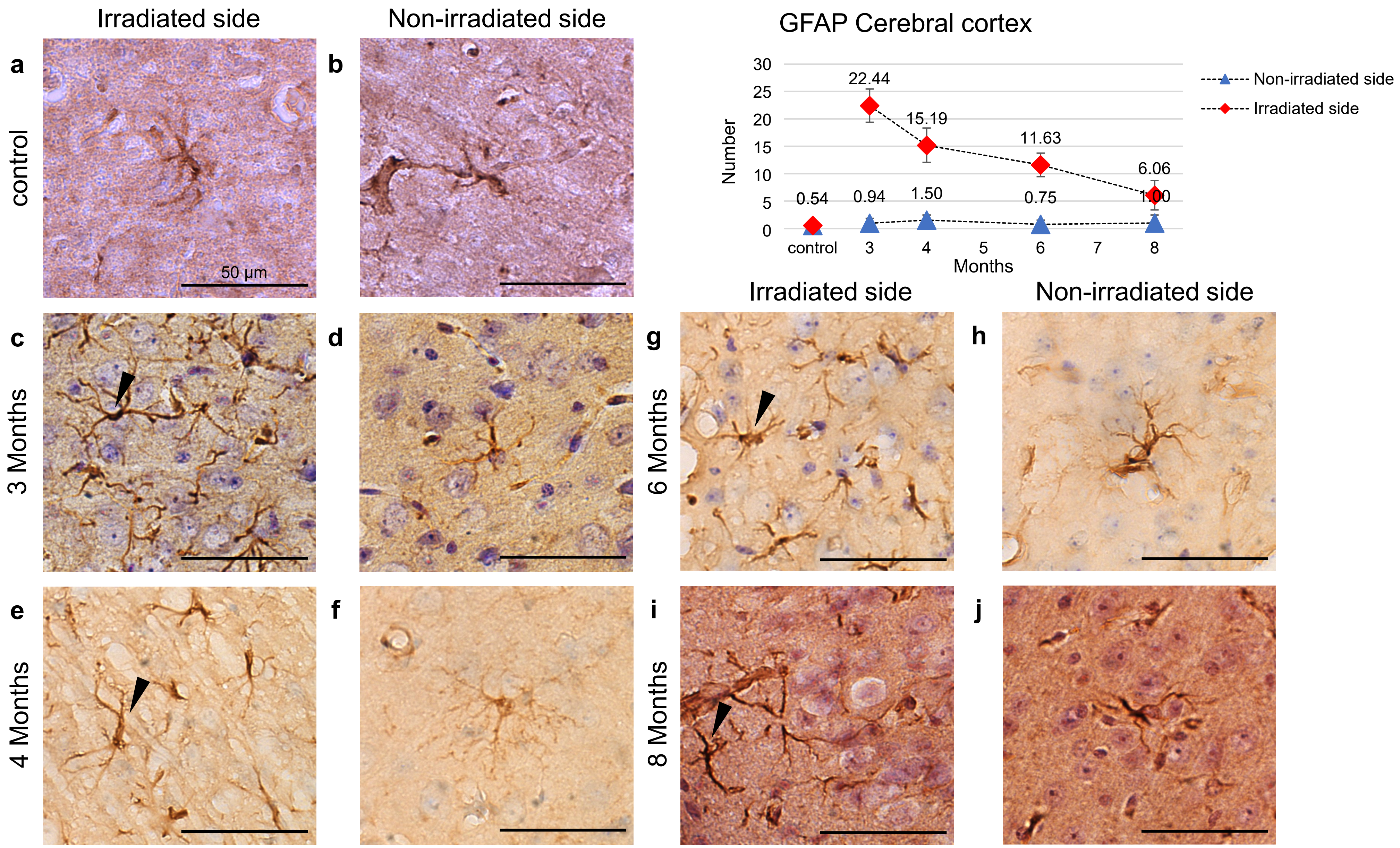

# Supplementary figure. 2B

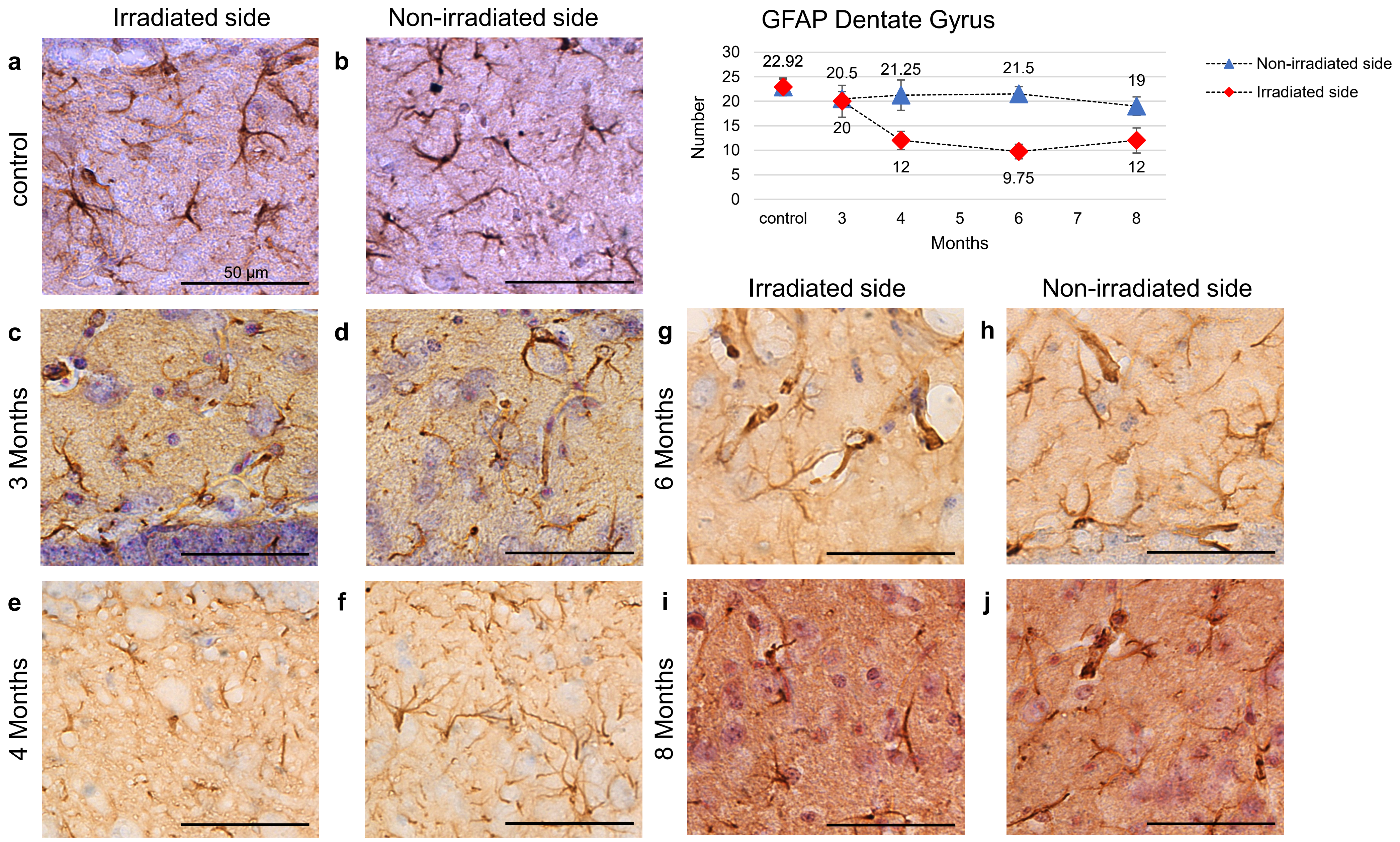

**Supplementary Figure1. Immunostaining of Iba-1 in RN model mice**

Histology of the cerebral cortex (A) and dentate gyrus (B) using ionized calcium binding adaptor molecule 1 (Iba-1) antibodies in irradiated and non-irradiated sides of the non-irradiated controls ($n$ = 3) (a, b), and brain radiation necrosis mice at 3 (c, d), 4 (e, f), 6 (g, h), 8 (i, j) months after irradiation ($n$ = 1, in each group). The graph indicates the numbers of microglia in each side. Scale bar = 50 μm. Arrowheads indicate microglia.

**Supplementary Figure 2. Immunostaining of GFAP in RN model mice**

Histology of the cerebral cortex (A) and dentate gyrus (B) using Glial fibrillary acidic protein (GFAP) antibodies in irradiated and non-irradiated sides of the non-irradiated controls ($n$ = 3) (a, b), and brain radiation necrosis mice at 3 (c, d), 4 (e, f), 6 (g, h), 8 (i, j) months after irradiation ($n$ = 1, in each group). The graph indicates the numbers of astrocytes in each side. Scale bar = 50 μm. Arrowheads indicate astrocytes.
